# Fine classification and phenological analysis of rice paddy based on multi-temporal general compact polarimetric SAR data

**DOI:** 10.3389/fpls.2024.1391735

**Published:** 2024-10-10

**Authors:** Xianyu Guo, Junjun Yin, Kun Li, Jian Yang

**Affiliations:** ^1^ School of Computer and Communication Engineering, University of Science and Technology Beijing, Beijing, China; ^2^ Aerospace Information Research Institute, Chinese Academy of Sciences, Beijing, China; ^3^ Department of Electronic Engineering, Tsinghua University, Beijing, China

**Keywords:** rice paddy, general compact polarimetric (CP) SAR, fine classification, phenological analysis, CTLR mode, π/4 mode

## Abstract

Fine classification and phenological information of rice paddy are of great significance for precision agricultural management. General compact polarimetric (CP) synthetic aperture radar (SAR) offers the advantage of providing rich polarimetric information, making it an important means of monitoring rice growth. Therefore, in response to the current challenges of difficulty in rice type classification and the small differences in phenological polarimetric characteristics, a novel strategy for fine classification and phenological analysis of rice paddy is proposed. This strategy thoroughly explores the polarimetric information of general CP SAR data and the target scattering characterization capabilities under different imaging modes. Firstly, the general CP SAR data is formalized using the standard CP descriptors, followed by the extraction of general CP features through the Δ*α_B_*/*α_B_* target decomposition method. optimal CP features are generated to achieve fine classification of rice paddy. Finally, 6 phenological stages of rice are analyzed based on the general CP features. The experiment results of rice classification show that the classification accuracy based on this strategy exceeds 90%, with a Kappa coefficient above 0.88. The highest classification accuracies were observed for transplanting hybrid rice paddy (T-H) and direct-sown japonica rice paddy (D-J), at 80.9% and 89.9%, respectively. The phenological evolution rule of the two rice types indicate that from early June (seedling stage) to late July (elongation stage), the CP feature variation trends of T-H and D-J are generally consistent. However, from October (mature stage) to November (harvest stage), the variation trends of the CP features for T-H and D-J are significantly different. The study found that from the booting-heading stage to the harvest stage, the linear π/4 mode outperforms circular and elliptical polarimetric modes in distinguishing different types of rice. Throughout the entire phenological period of rice growth, CP SAR of linear π/4 mode is surpasses that of other linear modes in discriminating different type of rice. The proposed strategy enables high-precision fine classification rice paddy, and the extracted general CP *α_B_* parameter effectively reflects the phenological change trends in rice growth.

## 1 Introduction

Rice is the primary staple crop worldwide, covering approximately 15% of the world’s total arable land. Furthermore, over 50% of the global population depends on rice as their primary food source (Maclean et al., 2002). Accurate information on the fine classification of rice paddies can enable precise and periodic estimation of rice yields. This information directly influences the government’s ability to formulate appropriate policies for grain production, distribution, storage, and transportation. Consequently, it serves as a crucial foundation for scientifically predicting and managing grain prices. Furthermore, rice phenology estimation is an important part of rice field management system (Mosleh et al., 2015). It not only helps field managers plan and implement different field management activities (such as irrigation, fertilization, etc.) in a timely manner, but also provides essential timing and crop growth references for yield estimates. In recent years, remote sensing technology has gradually replaced the traditional field observation method with its wide coverage and short revisiting period. Moreover, all-day and all-weather radar remote sensing has become an effective means of monitoring and estimating rice yield (Lopez-Sanchez et al., 2014). Fine classification and phenological analysis of rice paddies utilizing SAR data are particularly advantageous due to SAR’s capability to penetrate clouds and fog, enabling continuous monitoring of rice growth in all weather conditions. Furthermore, the characteristic parameters of polarimetric SAR are highly sensitive to the morphological structure and water content of the rice canopy, enhancing the accuracy of classification and analysis of rice paddies.

At present, the research on rice mapping based on fully polarimetric (FP) SAR data has gradually matured (Zhang et al., 2009; Yang et al., 2008; Li et al., 2014; Bouvet and Toan, 2011). As a new imaging radar system, CP SAR has emerged as one of the crucial development trends in the next generation of earth observation SAR systems. It transmits a polarization wave and receives two orthogonal polarization waves, which effectively reduces the complexity and energy consumption of SAR system and reduces the sensor volume. Currently, the most widely used CP SAR modes are π/4 mode (Souyris et al., 2005; Souyris and Mingot, 2002), DCP (Raney, 2006) and CTLR (Stacy and Press Austra, 2006; Raney, 2007). In agricultural radar remote sensing, rice classification and phenology estimation using CP SAR data is becoming a hotspot of current research (Yang et al., 2014; Deepika et al., 2015; Guo et al., 2021, Guo et al., 2022). However, all these studies are based on a single CP mode. Nowadays, there is no study in fine classification and phenology estimation of rice paddies based on the general CP mode. At present, there is a paucity of research focused on the fine classification and phenology estimation of rice paddies utilizing the general CP mode. Fortunately, our recent work (Yin et al., 2019) introduced a novel formalism for general CP SAR, paving the way for exploring the potential of CP SAR data from arbitrary electromagnetic wave transceiver modes in rice classification and phenology estimation.

Complex land-cover classification for FP SAR and CP SAR data primarily relies on intensity and polarimetric information. Various polarimetric decomposition methods are commonly employed to extract polarimetric characteristic parameters, enhancing the use of polarimetric information in classification tasks. Numerous polarimetric decomposition methods for the FP SAR data, have been developed based on target complexity. These methods include the two-component decomposition method based on Kennaugh matrix (Huynen, 1990; Holm and Barnes, 1988; Yang et al., 2006), decomposition methods using covariance matrix *C*_3_ or coherence matrix *T*_3_ based on scattering models (Freeman and Durden, 1998; Yamaguchi et al., 2005, Yamaguchi et al., 2006), eigenvector or eigenvalue analysis methods based on covariance matrix *C*_3_ or coherence matrix *T*_3_ (Cloude, 1985; Cloude and Pottier, 1996; van Zyl, 1993) and coherent decomposition methods based on scattering matrix *S* (Krogager, 1990; Cameron and Leung, 1990; Cameron and Rais, 2006; Touzi and Charbonneau, 2002). These decomposition methods are used to extract polarimetric features for land classification. Currently, there are two main polarimetric decomposition methods for CP SAR data. The first method involves reconstructing CP SAR into pseudo fully polarimetric SAR, followed by polarimetric decomposition using FP decomposition methods to extract polarimetric parameters. The second method involves directly performing polarimetric decomposition on the CP SAR data. Recently, few polarimetric decomposition methods have been developed for CP SAR, and most of them apply only to a single mode of compact polarization, such as *m*-*χ* decomposition (Raney et al., 2012), *m*-*δ* decomposition (Raney, 2007; Charbonneau et al., 2010) and *m*-*α_S_* decomposition (Cloude et al., 2012). However, these decomposition methods are only applicable to CP SAR of circular mode, and cannot be directly applied to CP SAR data in other modes without incident wave-based modification. We (Yin and Yang, 2020) developed a *α_S_-ϕ_s_* decomposition method, which expanded the *m*-*α_S_* decomposition method from circular polarization mode to π/4 mode. Therefore, we note that currently, few polarimetric decomposition methods can be directly applied to CP SAR of different modes as well as FP SAR data. We (Yin et al., 2016) developed a decomposition method applied to FP SAR data, achieving good results in identifying and separating target scattering mechanisms. Subsequently, we (Yin et al., 2019) modified the Δ*α_B_*/*α_B_* target decomposition method, enabling its application to CP SAR data in general CP mode.

Given the universality and robustness of Δ*α_B_*/*α_B_* method in both FP SAR and multi-mode CP SAR data, we introduced this method into the fine classification of multi-temporal rice paddy and phenological analysis of rice. Moreover, we the first time explored the ability of CP SAR data of arbitrary electromagnetic wave transceiver modes in rice classification and phenological analysis. We also extracted and analyzed the six temporal Δ*α_B_* and *α_B_* parameters of T-H and D-J based on FP SAR and general CP SAR. For fine classification of rice paddy, Δ*α_B_* and *α_B_* parameters were analyzed for distinguishing of T-H and D-J based on FP SAR data, CP SAR data of π/4 and CTLR modes. The Support Vector Machine (SVM) method was used to carry out a classification experiment based on the optimal Δ*α_B_* and *α_B_* parameters under the three kinds of SAR data (FP SAR, CP SAR of CTLR mode and π/4 mode). The classification results were then verified and evaluated. For phenological analysis of two types of rice, we analyzed the Δ*α_B_* and *α_B_* of general CP SAR across multiple phenological periods, including four CP modes: circular mode, linear π/4 mode and two kinds of ellipse modes. And we obtained the change rule of Δ*α_B_* and *α_B_* of two types of rice paddies in the phenological periods under multiple CP modes.

## 2 Study area and data

**Figure 1:**
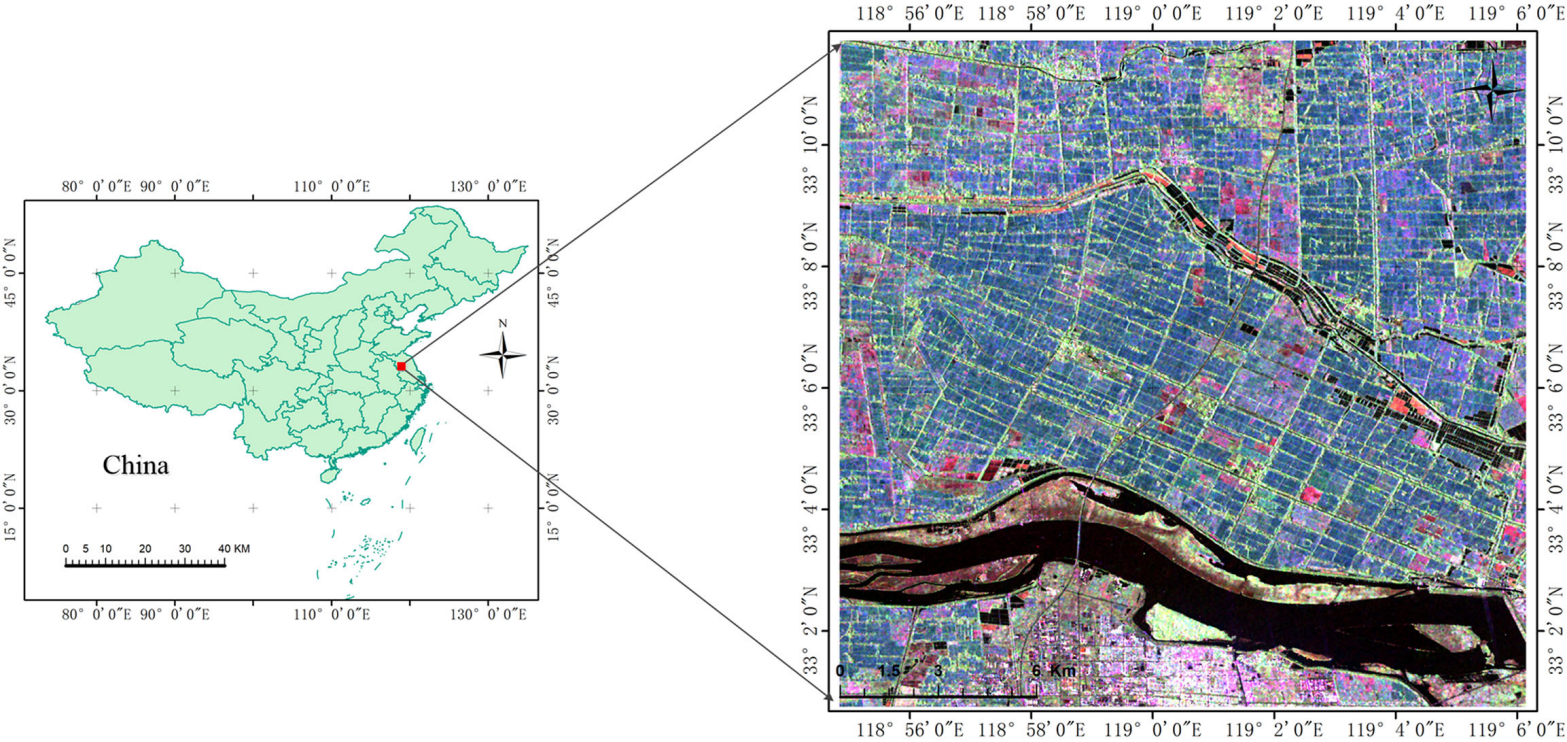
The color composite images [FP SAR VV (Red), VH (Green), and HH (Blue)] of the backscattering coefficients of FP SAR data on July 30, 2015.

**Figure 2:**
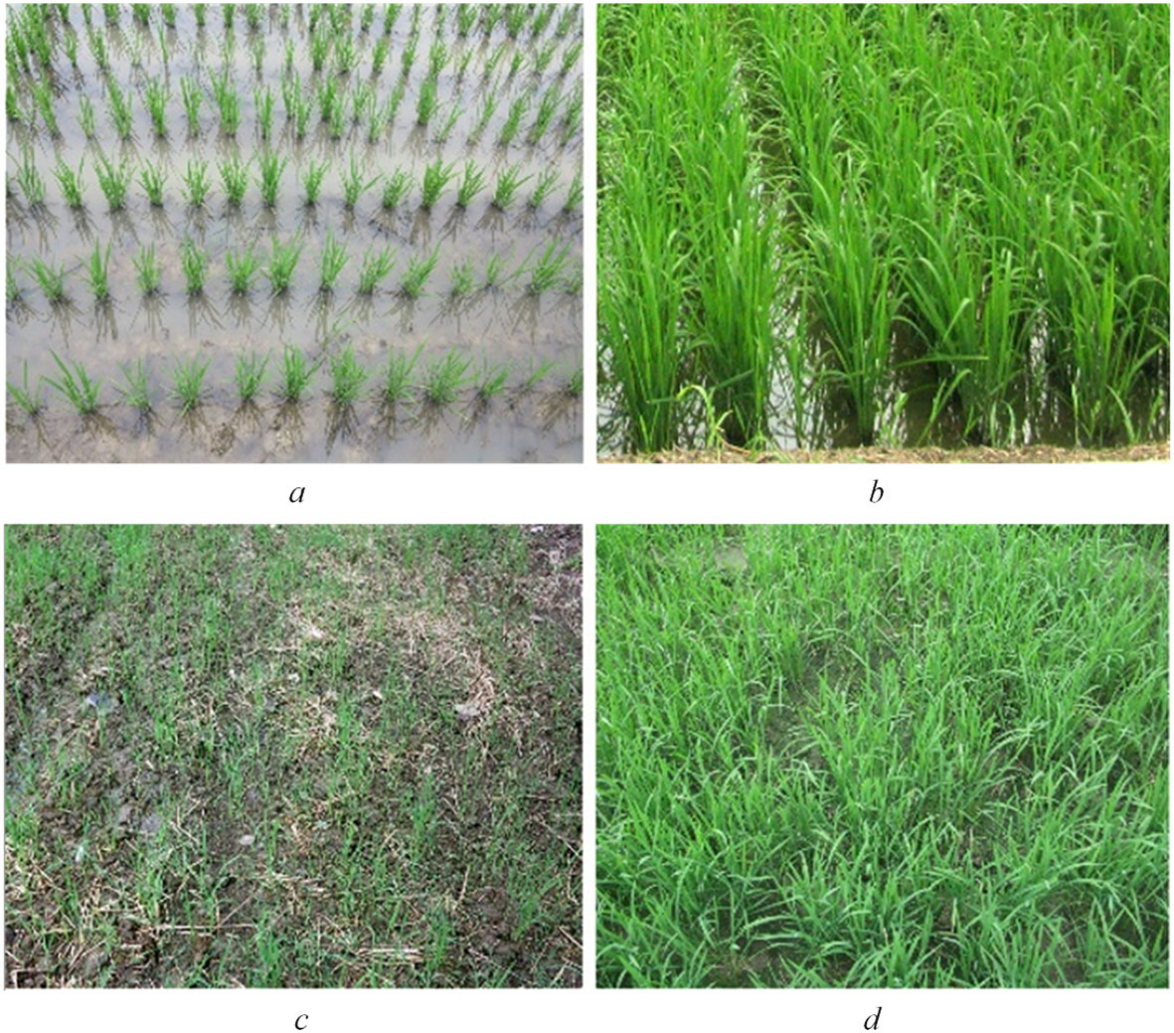
Field photos of two types of rice **(A)** Seedling stage of T-H; **(B)** Early tillering stage of T-H; **(C)** Seedling stage of D-J; **(D)** Early tillering stage of D-J).

Our research area is located around *Jinhu*, *Huai ‘an*, *Jiangsu*, *China*, covering approximately 40 km × 30 km (**Figure 1**). The central geographic coordinate of this area is *33°07 ‘05 “N*, *118°59′55.14″E*. The climate of the study area is classified as a subtropical temperate monsoon climate zone, with an average annual precipitation of 1085 mm. In terms of crops, the main crop in this area is rice with planting pattern once a year. Due to different planting methods, planting habits and rice varieties, this area mainly includes two types of rice paddies, namely, transplanting hybrid rice paddy (T-H) and direct-sown japonica rice paddy (D-J). In terms of transplanting paddy, rice seedlings need to be cultivated in nursery in advance, and then transplanted by artificial or mechanical transplanting. Besides, the row and pier spacing are approximately 30 cm and 15 cm. And, most of these rice seedlings are hybrid rice (e.g. ‘LIANGYOU-898’ and ‘XIEYOU-9308’) with growth cycle of about 120 days. In 2015, the T-H rice growth cycle spanned from mid-June to mid-October. For sowing paddy, rice seeds or small seedlings are directly sowed. Compared with the transplanting paddy, the rice seedlings had no obvious row-column rule with random uniform distribution. Meanwhile, these rice seeds or small seedlings are mostly japonica rice. (e.g. ‘HUAIDAO-5’ and ‘NANJING-9108’) with growth cycle of about 150 days. In 2015, the T-H rice growth cycle spanned from mid-June to early November. **Figure 2** shows field photos of two types of rice. We can see the difference between the two types of rice in the field photo in two phenological stages. In addition to the two types of rice paddies, the study area includes three other land cover types: urban, water and shoal naked land (SNL). Since the growth cycle of rice spans from June to November, we selected six temporal RADARSAT-2 C band FP SAR data. The FP SAR data parameters are shown in **Table 1**.

**Table 1:** FP SAR data parameters of multi-temporal RADARSAT-2.

Data acquisition date (Y/M/D)	DoY (Day of Year)	Imaging mode	Pixel Spacing (A × R, m)	Incidence Angle (deg)	Phenology Stage of Rice
2015/06/12 2015/07/30 2015/08/23 2015/09/16 2015/10/10 2015/11/03	163 211 235 259 283 307	FQ20W**^1^** FQ20W FQ20W FQ20W FQ20W FQ20W	5.2×7.6 5.2×7.6 5.2×7.6 5.2×7.6 5.2×7.6 5.2×7.6	38 – 41 38 – 41 38 – 41 38 – 41 38 – 41 38 – 41	Seedling Seedling–Elongation Booting–Heading Heading–Flowering Dough–Mature Harvest

**^1^** FQW, fine quad-polarimetry wide, 20 is the number of the beam position, which is related to the incidence angles.

Ground experiments were conducted as the satellite passed over the study area. High-precision GPS was used to collect the geographic coordinates of 42 rice parcels, including 28 T-H and 14 D-J parcels. Each parcel covers an area of more than 100 m × 100 m, ensuring sufficient pixel coverage. In addition, we also collected the geographic information of 8 water, 8 urban and 8 SNL parcels. In this study, the T-H, D-J, urban, SNL, and water parcels were divided into two groups: training and verification samples, based on their geographic coordinates. And the training and verification sets each accounted for 50% of total samples, with no overlap between the two.

## 3 Methodology

**Figure 3:**
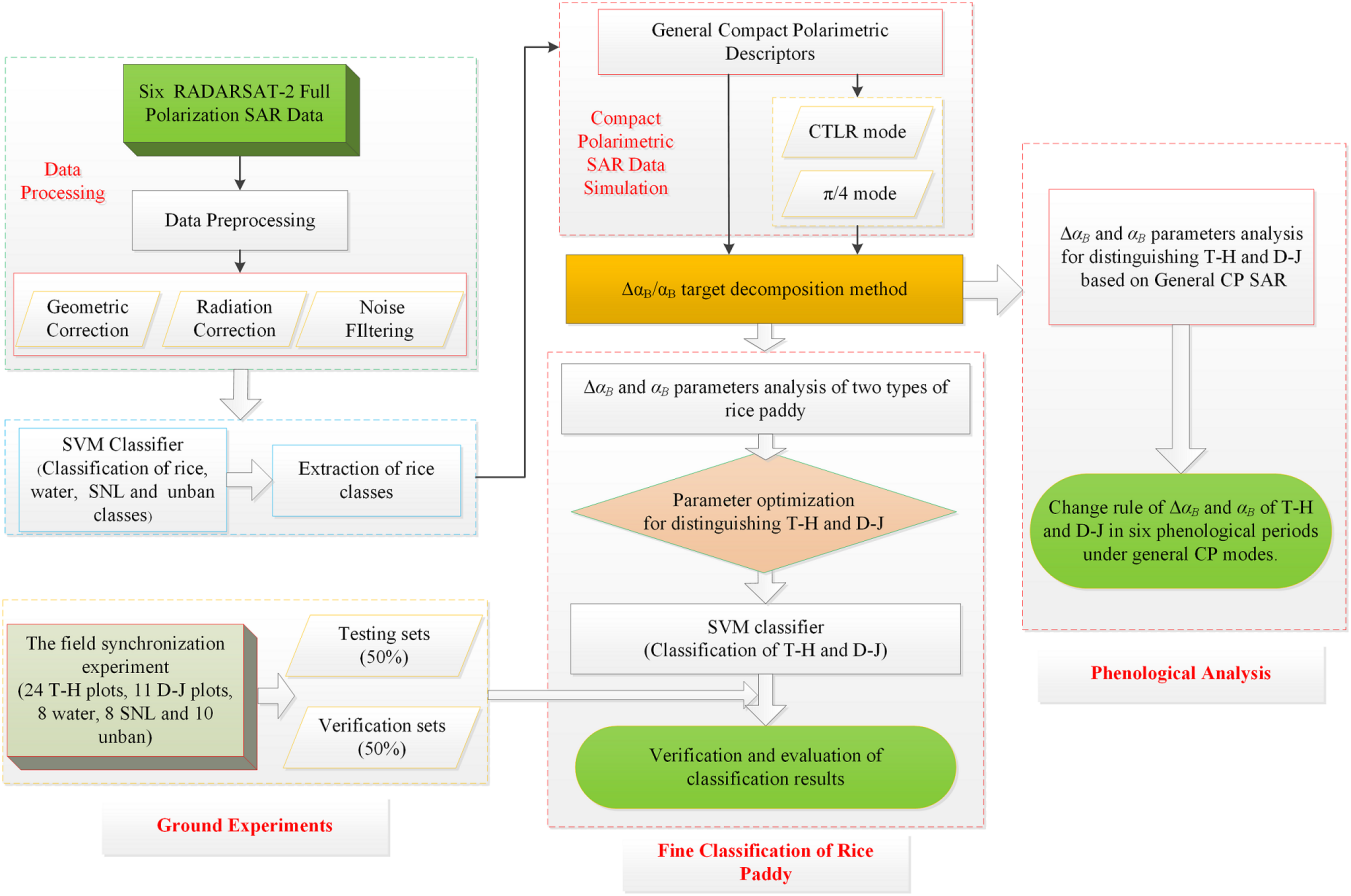
The specific flow chart.

First, six temporal FP RADARSAT-2 data were preprocessed, including radiometric correction, geometric correction and filtering. Next, utilizing the SVM method with FP SAR data, the study area was classified into four classes: rice, water, SNL, and unban. At the same time, we used general compact polarimetric descriptors to simulate the FP SAR data in the rice area, thereby obtaining general CP SAR data. Since our study focuses on the two types of rice paddies, we masked the rice class using the classification results of the SVM method. Then, the Δ*α_B_*/*α_B_* target decomposition method was introduced to carry out polarimetric decomposition for six temporal FP SAR data and general CP SAR data. Afterwards, using Δ*α_B_*/*α_B_* target decomposition method on general CP SAR data, we performed fine classification and phenological analysis of rice paddies. For fine classification of rice paddy, Δ*α_B_* and *α_B_* parameters were analyzed for distinguishing of T-H and D-J based on FP SAR data, CP SAR data of π/4 and CTLR modes, and the SVM method was used to carry out a classification experiment based on the optimal Δ*α_B_* and *α_B_* parameters under the three kinds of SAR data (FP SAR, CP SAR of CTLR mode and π/4 mode). Finally, the classification results were verified and evaluated. Moreover, for phenological analysis, we analyzed the Δ*α_B_* and *α_B_* of general CP SAR, including four CP modes (circular mode, linear π/4 mode and two kinds of ellipse modes) across multiple phenological periods for both types of rice paddies. We then obtained the change rule of Δ*α_B_* and *α_B_* for two types of rice paddies throughout the phenological periods under multiple CP modes. **Figure 3** shows the specific flow chart of the methodology.

Specifically, for preprocessing work (radiometric correction, geometric correction and filtering), the detailed parameters information of radiometric correction provided by the header file of FP SAR data are used for radiometric correction. Then the SAR image is speckle filtered using a 7×7 Lee filter. For all RADARSAT-2 FP SAR data, we extract the complex scattering matrix *S* based on PolSARpro software (version 6.0, https://step.esa.int/main/toolboxes/polsarpro-v6-0-biomass-edition-toolbox/). And, the preprocessing work is carried out in ENVI image processing software (version 5.3, https://www.cnblogs.com/enviidl/p/16275745.html) and PolSARpro v6.0. For SVM method, the algorithm parameters used in this study are introduced in detail in Section 3.3. Moreover, the general compact polarimetric SAR descriptors and Δ*α_B_*/*α_B_* target decomposition method are programmed in matlab software (version R2021b, https://ww2.mathworks.cn/en/products/matlab.html).

### 3.1 Polarimetric features

#### 3.1.1 π/4 mode and CTLR mode

We simulated CP SAR data using FP SAR data based on π/4 mode and CTLR mode respectively. For π/4 mode, this mode transmits linear polarization waves in a 45°C direction, receiving horizontal and vertical polarization echo signals (Souyris et al., 2005; Souyris and Mingot, 2002; Wang et al., 2018). ${\vec{\text{k}}}_{\pi /4}$ , scattering vector under π/4 mode, is expressed as


(1) $${\vec{\text{k}}}_{\pi /4}=\frac{1}{\sqrt{2}}{\left[\begin{array}{cc} S_{\text{HH}}+S_{\text{HV}} & S_{\text{VV}}+S_{\text{HV}} \end{array}\right]}^{T}$$

where, *S*_HH_, *S*_HV_ and *S*_VV_ are three elements of Sinclair matrix.

For CTLR mode, this mode transmits right circular polarization and receives horizontal and vertical polarization echo signals (Raney, 2006, Raney, 2007; Cloude et al., 2012; Wang et al., 2018). ${\vec{\text{k}}}_{\text{CTLR}}$ , scattering vector under CTLR mode, is expressed as


(2) $${\vec{\text{k}}}_{\text{CTLR}}=\frac{1}{\sqrt{2}}{\left[\begin{array}{cc} S_{\text{HH}}-iS_{\text{HV}} & S_{\text{HV}}-iS_{\text{VV}} \end{array}\right]}^{T}$$

The Jones coherency matrix (*C*_2_) of CP SAR, that is, the second-order statistic of the scattering vector, can be expressed as follows:


(3) $$C_{2}=\langle {\vec{\text{k}}}_{CP}{\vec{\text{k}}}_{CP}^{*T}\rangle$$

where, ${\vec{\text{k}}}_{CP}$ is scattering vector of CP SAR.

For full polarimetric SAR, the radar transmits horizontal and vertical polarization waves, and receives horizontal and vertical polarization waves. In the single-station backscattering system, the three-dimensional target vector k is expressed as


(4) $$\vec{\text{k}}=\frac{1}{\sqrt{2}}{\left[\begin{array}{ccc} S_{\text{HH}}+S_{\text{VV}} & S_{\text{HH}}-S_{\text{VV}} & 2S_{\text{HV}} \end{array}\right]}^{T}$$

The full polarization coherence matrix *T* can be expressed as


(5) $$T_{3}=\left[\begin{array}{ccc} T_{11} & T_{12} & T_{13}\\ T_{21} & T_{22} & T_{23}\\ T_{31} & T_{32} & T_{33} \end{array}\right]=\langle {\to \vec{\text{k}}\vec{\text{k}}}^{*T}\rangle$$

where, T_*_ is element of coherence matrix *T*.

#### 3.1.2 General compact polarimetric descriptors

The electromagnetic field is usually expressed in the form of a polarization ellipse, which contains two parameters, namely, the ellipticity angle *χ and* the orientation angle *θ* of the ellipse.


(6) $$\begin{array}{l} {\vec{E}}_{i}\;(\theta ,\chi )=\left[\begin{array}{c} a\\ b \end{array}\right]=\left[\begin{array}{cc} cos\theta & -sin\theta \\ sin\theta & cos\theta \end{array}\right]\left[\begin{array}{c} cos\chi \\ jsin\chi \end{array}\right]\\ =\left[\begin{array}{c} cos\theta cos\chi -jsin\theta sin\chi \\ sin\theta cos\chi +jcos\theta sin\chi \end{array}\right] \end{array}$$

where, *a* and *b* are transmitting wave elements and ${\left|a\right|}^{2}+{\left|b\right|}^{2}=1$ . For the arbitrary transmitting wave ${\vec{E}}_{i}(\theta ,\chi )$ , the received wave ${\vec{E}}_{r}\;(\theta ,\chi )$ can be expressed as follows:


(7) $$\begin{array}{l} {\vec{E}}_{r}\;(\theta ,\chi )=S\;{\vec{E}}_{i}\;(\theta ,\chi )\\ =\left[\begin{array}{cc} S_{HH} & S_{HV}\\ S_{VH} & S_{VV} \end{array}\right]=\left[\begin{array}{c} aS_{HH}+bS_{HV}\\ bS_{VV}+aS_{VH} \end{array}\right] \end{array}$$

In the Equation 7, ${\vec{E}}_{r}\;(\theta ,\chi )$ is the representation of H/V polarized basis ${\vec{E}}_{r}\;(\theta ,\chi )={\left[\begin{array}{cc} E_{HC} & E_{VC} \end{array}\right]}^{T}$ , including $E_{HC}=aS_{HH}+bS_{HV}$ , $E_{VC}=bS_{VV}+aS_{VH}$ . *T* is matrix transpose. $E_{HC}$ contains $S_{HH}$ and $E_{VC}$ contains $S_{VV}$ . The backscattering characteristics of the target are mainly retained in the co-polarization ratio. When *a*≠0 and *b*≠0, the backscattering vector can be described


(8) $$\begin{array}{l} \left[\begin{array}{c} E_{1}\\ E_{2} \end{array}\right]=\left[\begin{array}{cc} a^{-1} & 0\\ 0 & b^{-1} \end{array}\right]{\vec{E}}_{r}\;(\theta ,\chi )\\ =\left[\begin{array}{c} S_{HH}+\frac{b}{a}S_{HV}\\ S_{VV}+\frac{a}{b}S_{VH} \end{array}\right] \end{array}$$

where *E*_1_ and *E*_2_ are normalized elements of ${\vec{E}}_{r}\;(\theta ,\chi )$ to represent the characteristics of backscattering waves. From Equation 8, we can form two new CP vectors ( ${\vec{\text{k}}}_{1}$ and ${\vec{\text{k}}}_{2}$ )


(9) $${\vec{\text{k}}}_{1}={\left[\begin{array}{cc} E_{1} & E_{2} \end{array}\right]}^{T}$$

(10) $${\vec{\text{k}}}_{2}={\left[\begin{array}{cc} E_{1}+E_{2} & E_{1}-E_{2} \end{array}\right]}^{T}/\sqrt{2}$$

The corresponding second-order statistic of the scattering vector, namely the covariance matrix *C*_2_ and coherence matrix *T*_2_ of the normalized vector, are used to describe the partially polarized scattered waves


(11) $$C_{2}=\langle \overset{⇀}{\underset{1}{\text{k}}}\vec{\text{k}}{*}_{1}^{T}\rangle =\left[\begin{array}{cc} \langle {\left|E_{1}\right|}^{2}\rangle & \langle E_{1}E_{2}^{*}\rangle \\ \langle E_{2}E_{1}^{*}\rangle & \langle {\left|E_{2}\right|}^{2}\rangle \end{array}\right]$$

(12) $$T_{2}=\langle \overset{⇀}{\underset{2}{\text{k}}}\vec{\text{k}}{*}_{2}^{T}\rangle =\left[\begin{array}{cc} \frac{\langle {\left|E_{1}+E_{2}\right|}^{2}\rangle }{2} & \frac{\langle (E_{1}+E_{2}){\left(E_{1}-E_{2}\right)}^{*}\rangle }{2}\\ \frac{\langle (E_{1}-E_{2}){\left(E_{1}+E_{2}\right)}^{*}\rangle }{2} & \frac{\langle {\left|E_{1}-E_{2}\right|}^{2}\rangle }{2} \end{array}\right]$$

where 〈⋅〉 denotes the ensemble average. ^*T^ represents matrix conjugate transpose. The general CP SAR descriptor vector in Equation 8 (or the covariance *C*_2_ and coherence matrix *C*_2_ defined in Equations 11, 12) provides a unified method for scattering analysis of CP SAR data.

### 3.2 Δ*α_B_*/*α_B_* target decomposition method based on FP SAR and CP SAR

#### 3.2.1 Δ*α_B_*/*α_B_* target decomposition method based on FP SAR

The Δ*α_B_*/*α_B_* target decomposition method is mainly based on the average physical scattering mechanism to solve the scattering inconsistency and dominant scattering mechanism. We defined a new parameter, which is rotation invariant (Yin et al., 2016).


(13) $$\alpha_{B}=\arctan\left(\frac{T_{22}+T_{33}}{T_{11}}\right)$$

where *T*_11_, *T*_22_, *T*_33_ are the main diagonal elements of an arbitrary backscattering coherence matrix *T* (see Equation 5)*. α_B_* is a function of the co-polarized channel ratio and the co-polarized channel correlation. For the single look case, *α_B_* is only related to the co-polarized channel ratio. Thus, in order to measure the randomness inherent in the multi-look case, another parameter Δ*α_B_* is expressed as


(14) $$\Delta \alpha_{B}=\alpha_{B}-\alpha_{0}$$

where,


(15) $$\alpha_{0}=\arctan\left(\frac{{\left|\rho_{r}-1\right|}^{2}}{{\left|\rho_{r}+1\right|}^{2}}\right)$$

with,


(16) $$\rho_{r}=\left|\rho_{r}\right|e^{j\phi_{r}}=\sqrt{\frac{\langle {\left|S_{VV}\right|}^{2}\rangle }{\langle {\left|S_{HH}\right|}^{2}\rangle }}\;e^{j\left(\langle \phi_{VV}-\phi_{HH}\rangle \right)}$$

where |*ρ_r_* | and |*ϕ_r_* |are the average copolarization amplitude ratio and the average copolarization phase difference, respectively. *ϕ_VV_ and ϕ_HH_* are the phases of *VV* and *HH* polarization, respectively*. α_B_* is used to describe the physical scattering mechanism, and Δ*α_B_* describes the scattering randomness.

#### 3.2.2 Δ*α_B_/α_B_* target decomposition method based on CP SAR

For the CP SAR data, from the *T*_2_ matrix, the parameter *α_BCP_* was defined as (Yin et al., 2019)


(17) $$\alpha_{BCP}=\arctan\left(\frac{{\left|E_{1}-E_{2}\right|}^{2}}{{\left|E_{1}+E_{2}\right|}^{2}}\right)$$

where, $\alpha_{BCP}\in \left[\begin{array}{cc} 0{}^{\circ} & 90{}^{\circ} \end{array}\right]$ , is used to describe the average scattering mechanism. *E*_1_, and *E*_2_ are the formalized elements of ${\vec{E}}_{r}\;\left(\begin{array}{cc} \theta , & \chi \end{array}\right)$ to characterize the backscattered wave. For the general compact polarization, $E_{1}=S_{HH}+\frac{b}{a}S_{HV}$ and $E_{2}=S_{VV}+\frac{a}{b}S_{VH}$ (see Equation 8).

In deterministic scattering without rotation, the cross-polarization quantity *S_HV_* is typically smaller than the co-polarization quantity. In surface scattering region, such as the water surface, *α_BCP_* is close to 0°. In regions dominated by double-bounce scattering, *α_BCP_* approaches 90°while in areas characterized by volume scattering, *α_BCP_* around 45°.

Since *α_BCP_* can be expanded further,


(18) $$\alpha_{BCP}=\arctan\left(\frac{{\left|1-\rho_{\text{cp}}\right|}^{2}+2\left|\rho_{\text{cp}}\right|\cos\phi_{\text{cp}}(1-\left|\rho_{\text{cp}}\right|)}{{\left|1+\rho_{\text{cp}}\right|}^{2}-2\left|\rho_{\text{cp}}\right|\cos\phi_{\text{cp}}(1-\left|\rho_{\text{cp}}\right|)}\right)$$

where, functions of channel ratio *ρ*_cp_, channel correlation coefficient *r*_cp,_ and phase difference *ϕ*_cp_ was defined as


(19) $$\begin{array}{l} \rho_{\text{CP}}=\sqrt{\frac{\langle {\left|E_{2}\right|}^{2}\rangle }{\langle {\left|E_{1}\right|}^{2}\rangle }}\;e^{j\text{angle}\left(\langle E_{2}E_{1}^{*}\rangle \right)}\;\\ r_{\text{CP}}=\frac{\langle E_{1}E_{2}^{*}\rangle }{\sqrt{\langle {\left|E_{1}\right|}^{2}\rangle \langle {\left|E_{2}\right|}^{2}\rangle }},\phi_{\text{CP}}=\text{angle(}\langle E_{2}E_{1}^{*}\rangle ) \end{array}$$

|*r*_cp_| is the main index to describe the random backscattering process. When the |*r*_cp_| is close to 1, it indicates that a coherent scattering and *α*_BCP_ is determined by channel ratio *ρ*_CP_. When the |*r*_cp_| is close to 0, it indicates that backscattering comes from randomly distributed scattering objects. Meanwhile, *α*_BCP_ is close to 45°.

Besides, similar to Δ*α_B_*, a physical parameter is defined to describe the scattering incoherence of the target. Δ*α_BCP_* is expressed as


(20) $$\Delta \alpha_{BCP}=\alpha_{BCP}-\alpha_{0CP}$$

where,


(21) $$\alpha_{0CP}=\arctan\left(\frac{{\left|\rho_{\text{CP}}-1\right|}^{2}}{{\left|\rho_{\text{CP}}+1\right|}^{2}}\right)$$

$\alpha_{BCP}$ is used to describe the average scattering mechanism. *α_0CP_* can be regarded as an ideal scattering mechanism determined only by the average polarization ratio. And *ρ*_CP_ is channel ratio.

In general, Δ*α_B_* and Δ*α_BCP_* can describe the scattering randomness of the target, with their symbols determined by the phase difference in the co-polarization channel. If in a resolution cell, all scattering objects have the same scattering mechanism and the orientation angle is consistent with the dielectric constant, the co-polarization correlation coefficient *r*_cp_ and *r*_c_ is high, resulting in Δ*α_B_* and Δ*α_BCP_* close to 0°. For the double-bounce scattering process, because their physical models are mainly described by the characteristics of phase difference (±π) of the co-polarization channel, the values of Δ*α_B_* and Δ*α_BCP_* should be less than 0°. In contrast, for single scattering and volume scattering, because their physical models are mainly described by the characteristics of phase difference (±π/2) of the co-polarization channel, the values of Δ*α_B_* and Δ*α_BCP_* should be greater than 0.

### 3.3 SVM classification method

The SVM is a powerful machine learning algorithm used for various applications, including classification analysis, regression analysis, and pattern recognition (Suykens and Vandewalle, 1999; Huang et al., 2012). The idea of the SVM classification method is to enable the optimal hyperplane to have the maximum classification interval. For this study, we used the Radial Basis Function (RBF) kernel in the SVM classifier. Meanwhile, the parameter of RBF kernel function (Gamma) controls the influence distance of a single training point. A small Gamma results in a smaller influence, while a large Gamma increases the influence range. In our experiments, we set the Gamma parameter as the reciprocal of the input parameter. Besides, the penalty parameter (*C*) is the penalty parameter, the tolerance for error. The higher *C* is, the less error is tolerated and the easier it is to overfit. The smaller *C* is, the less fit it is. If *C* is too large or too small, the generalization ability becomes worse. In the experiment, *C* is set to 100. It should be noted that the spatial resolution of the original image is used for classification, and the classification probability threshold is 0.

## 4 Experiment and discussion

First, we used FP SAR data to divide the study into 4 classes based on SVM classification method, namely rice, water, urban and SNL classes. We then focused exclusively on the rice regions for further analysis. Next, we utilized FP SAR, as well as CP SAR in π/4 and CTLR modes, and general CP SAR data to extract the six temporal Δ*α_B_* and *α_B_* parameters of rice paddy using Δ*α_B_* and *α_B_* method.

The experiment is conducted on a computer equipped with an Intel Core i7-9750H processor (6 cores, 2.60 GHz), 16 GB of DDR4 RAM, an NVIDIA Quadro GPU, and running Windows 10. The image pixel size in this study is 2000 × 2000, and the area covered by the study region is approximately 1200 square kilometers. Based on the proposed method, we obtained experimental results and recorded the algorithm’s running time (approximately 38 minutes and 16 seconds). Therefore, under this research scenario (pixel size: 2000 × 2000), the computational efficiency for rice classification and phenological analysis is acceptable. In addition, the present parallel computing method can also improve the computational efficiency of the proposed method to a certain extent.

### 4.1 Δ*α_B_* and *α_B_* parameters analysis of two types of rice paddies based on FP SAR data, CP SAR data of π/4 and CTLR modes

**Figure 4:**
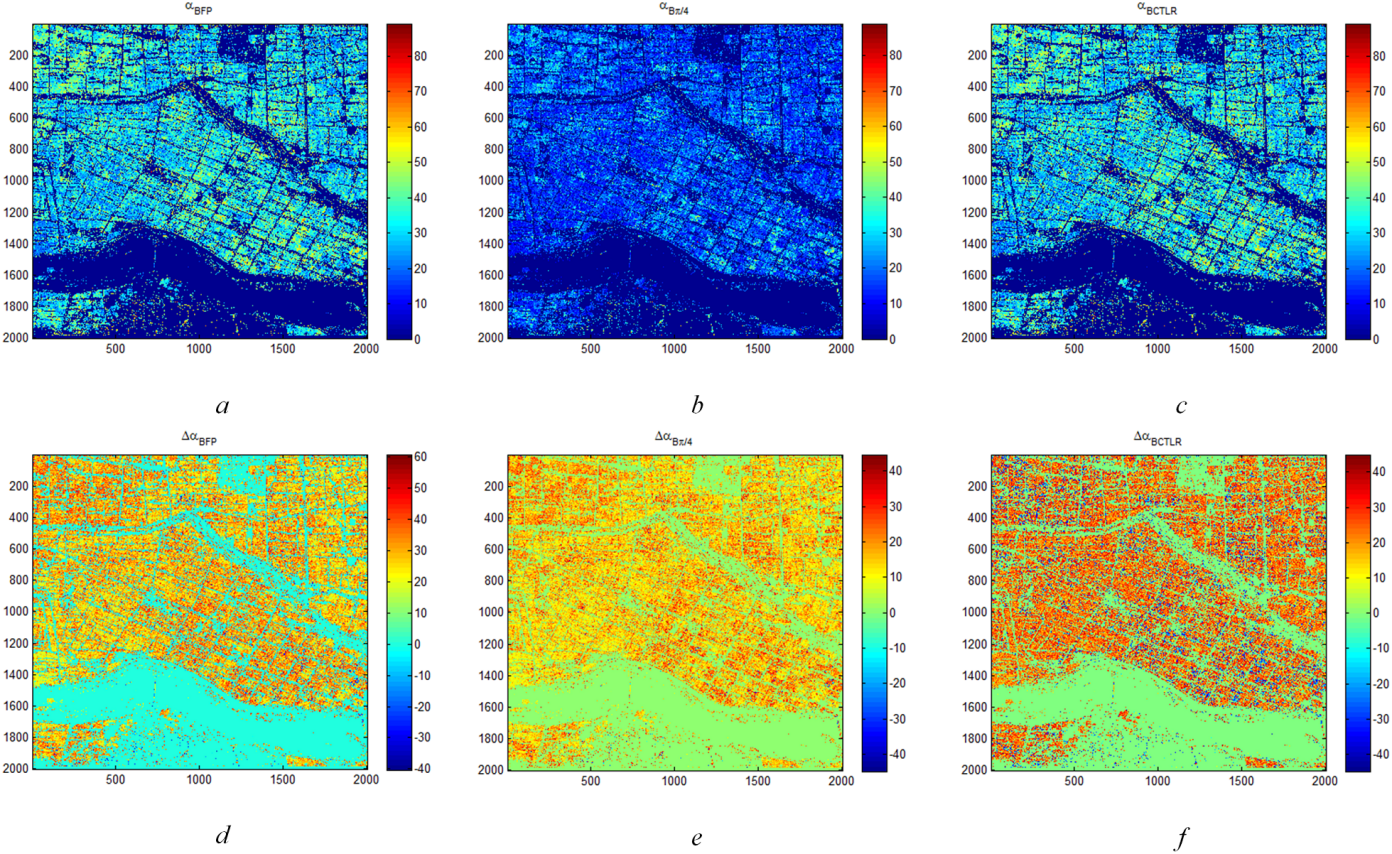
Δ*α_B_* and *α_B_* parameters of rice region based on FP SAR data, CP SAR data of π/4 mode and CTLR mode on June 12 **(A)** is *α_B_* and **(D)** is Δ*α_B_* based on FP SAR data; **(B)** is *α_B_* and **(E)** is Δα*_B_* based on CP SAR data of π/4 mode; **(C)** is *α_B_* and **(F)** is Δ*α_B_* based on CP SAR data of CTLR mode).

According to CP SAR theory in Sections 3.1.1 and 3.1.2, CP SAR data of π/4 and CTLR modes are two typical and widely applied CP mode. Therefore, we first carried out Δ*α_B_* and *α_B_* parameters analysis of two types of rice paddies based on FP SAR data, CP SAR data of π/4 and CTLR modes. **Figure 4** shows Δ*α_B_* and *α_B_* parameters of rice region based on FP SAR data, CP SAR data of π/4 and CTLR modes on June 12. As noted in Section 3.2, the αB range spans from 0° to 90°, indicating regions predominantly influenced by surface scattering, volume scattering and double-bounce scattering respectively. **Figures 4A–C** show that there are essentially two states of *α_B_* in the rice region: one greater than 45° and the other less than 45°. On June 12, the two types of rice were basically in the seedling stage, and the paddy in the seedling stage showed more information about the underlying surface of the paddy on the SAR image. Besides, the underlying surface of T-H is water surface, which is prone to surface scattering. In contrast, the underlying surface of D-J is moist soil. Therefore, it is obvious that the surface scattering component of D-J is smaller than that of T-H. Furthermore, most regions with high *α_B_* values are D-J paddy, while the region with low *α_B_* values area T-H paddy. In addition, there is one situation. Since T-H has a shorter growth period than D-J, it is possible that some farmers have not completed transplanting in this period, leading to the bare land in this area. Consequently, in addition to D-J paddy, some areas with high *α_B_* values might be T-H paddy where transplanting was not yet completed.

For Δ*α_B_* parameter, when Δ*α_B_* is greater than 0, it shows more surface scattering and volume scattering of ground objects. Conversely, when Δ*α_B_* is less than 0, it shows more double-bounce scattering. Furthermore, as shown in **Figures 4D–F**, Δ*α_B_* in the rice region is basically less than 0. In some areas, the Δ*α_B_* based on CP SAR data of CTLR mode exceeds 0, indicating predominant double-bounce scattering. These regions are more likely to be D-J.

With the growth of rice plants, the underlying surface information is covered, which results in representing more vegetation information in radar images. on June 12 and August 23, the Δ*α_B_* and *α_B_* are relatively uniform in the rice region. Therefore, Δ*α_B_* and *α_B_* parameters based on FP, CP of CTLR and CP of π/4 SAR data respectively, could not distinguish between T-H and D-J in these two periods.

**Figure 5:**
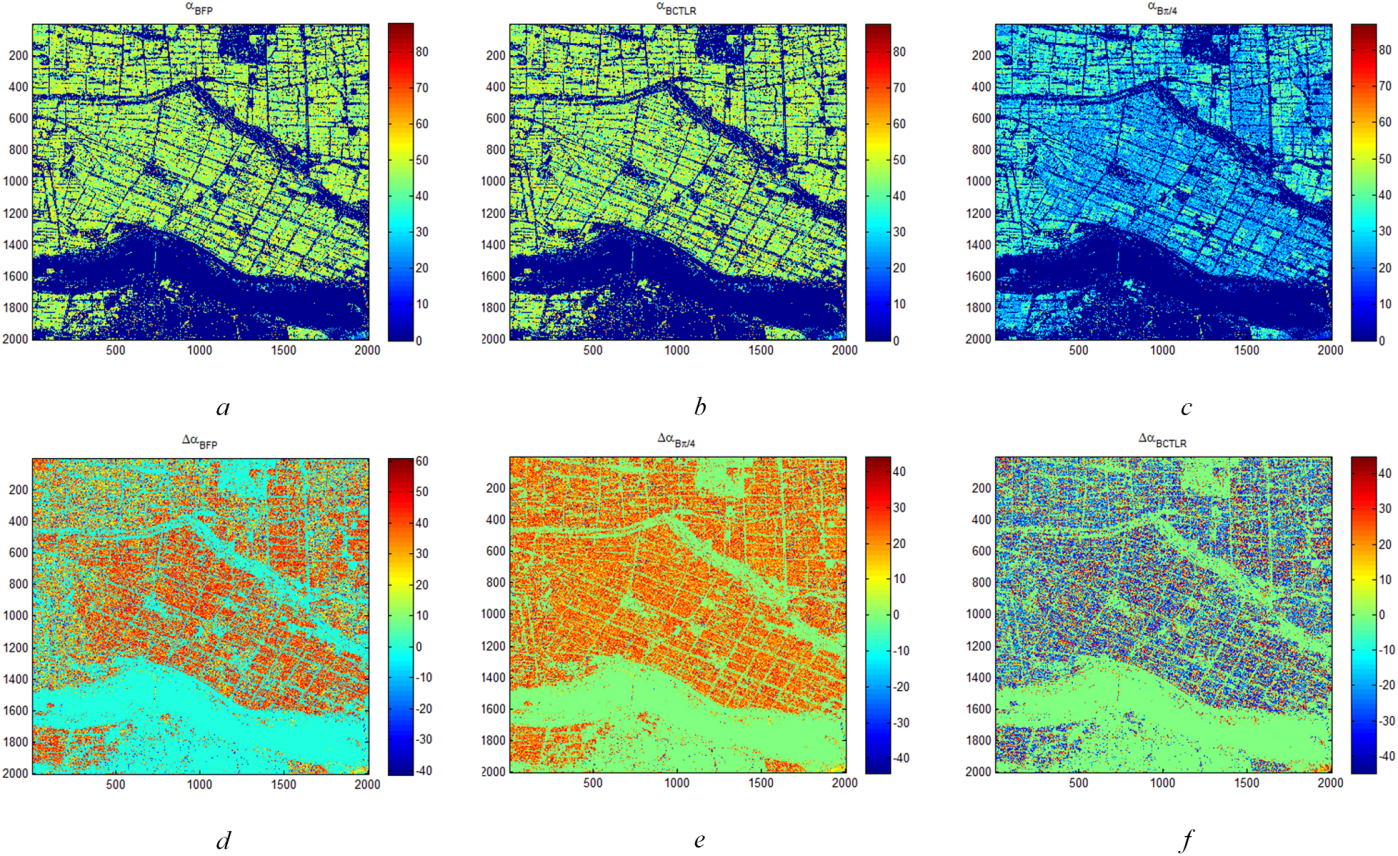
Δα*_B_* and α*_B_* parameters of rice region based on FP SAR data, CP SAR data of π/4 mode and CTLR mode on September 16 **(A)** is α*_B_* and **(D)** is Δα*_B_* based on FP SAR data; **(B)** is α*_B_* and **(E)** is Δα*_B_* based on CP SAR data of π/4 mode; **(C)** is α*_B_* and **(F)** is Δα*_B_* based on CP SAR data of CTLR mode).

**Figure 5** shows Δ*α_B_* and *α_B_* parameters of rice region based on FP SAR data, CP SAR data of π/4 mode and CTLR mode on September 16. On September 16, the phenology stage of rice is Heading–Flowering stage (**Table 1**). Therefore, the information of ear of rice is presented in this period. Due to the short period of T-H, the ear of rice of T-H grow earlier and are thicker than that of D-J, which makes the surface scattering of T-H larger than that of D-J. Additionally, D-J is larger than T-H in *α_B_* parameter. Comparing **Figures 5A–C**, we can find that in distinguishing the two types of rice paddies, CP SAR data of π/4 mode data is better than CP SAR data of CTLR mode data and FP SAR data in this period. For Δ*α_B_* parameters (**Figures 5D–F**), it’s almost impossible to see the difference between the two types of rice paddies. On October 10, the phenology stage of rice is Dough–Mature stage (**Table 1**). In this period, the rice ears of both T-H and D-J had mostly developed and begun to mature, leading to similar radar signatures for the rice panicles. Therefore, surface scattering, volume scattering and double-bounce scattering are similar.

**Figure 6:**
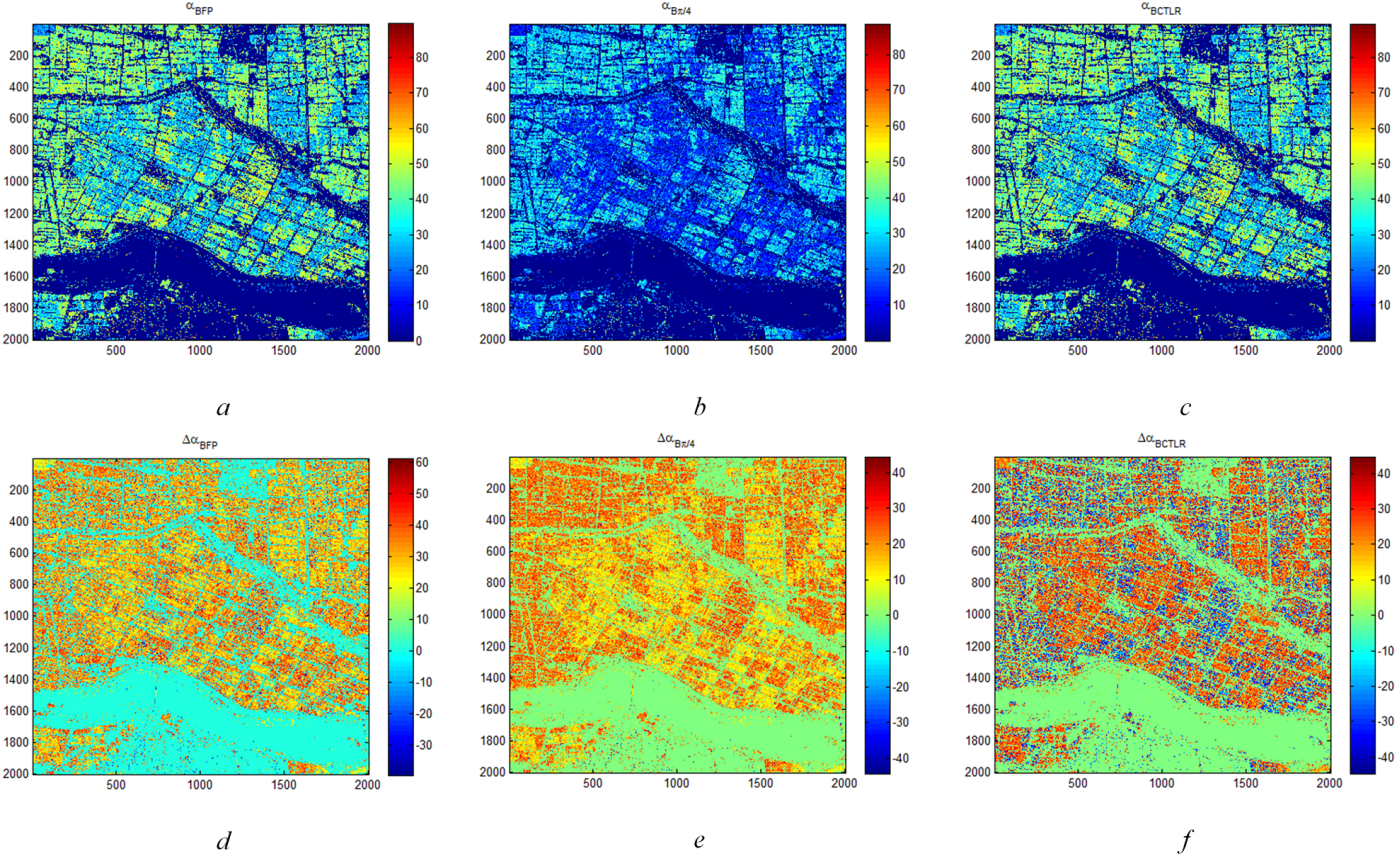
Δα*_B_* and αB parameters of rice region based on FP SAR data, CP SAR data of π/4 mode and CTLR mode on November 3 **(A)** is α*_B_* and **(D)** is Δα*_B_* based on FP SAR data; **(B)** is α*_B_* and **(E)** is Δα*_B_* based on CP SAR data of π/4 mode; **(C)** is α*_B_* and **(F)** is Δα*_B_* based on CP SAR data of CTLR mode).

**Figure 6** shows Δ*α_B_* and *α_B_* parameters of rice region based on FP SAR data, CP SAR data of π/4 mode and CP SAR data of CTLR mode on November 3. On November 3, the phenology stage of rice is Harvest stage (**Table 1**). Since the growth cycle of T-H is shorter than that of D-J, most of T-H had been harvested during this period. Therefore, the underlying soil of T-H is exposed naked, which shows soil characteristics in radar images. However, D-J has a long growth cycle. In this period, rice is in the mature stage and has not been harvested. Thus, the radar images capture the characteristics of the rice plants. Therefore, Δ*α_B_* and *α_B_* images show obvious differences between the two types of rice paddies. As shown in **Figures 6A–C**, that the values of *α_B_* of D-J are larger than those of T-H. Similarly, for **Figures 6D–F**, the values of Δ*α_B_* of D-J are also significantly different from those of T-H. However, the Δ*α_B_* and *α_B_* images show that *α_B_* is more stable than Δ*α_B_*.

**Figure 7:**
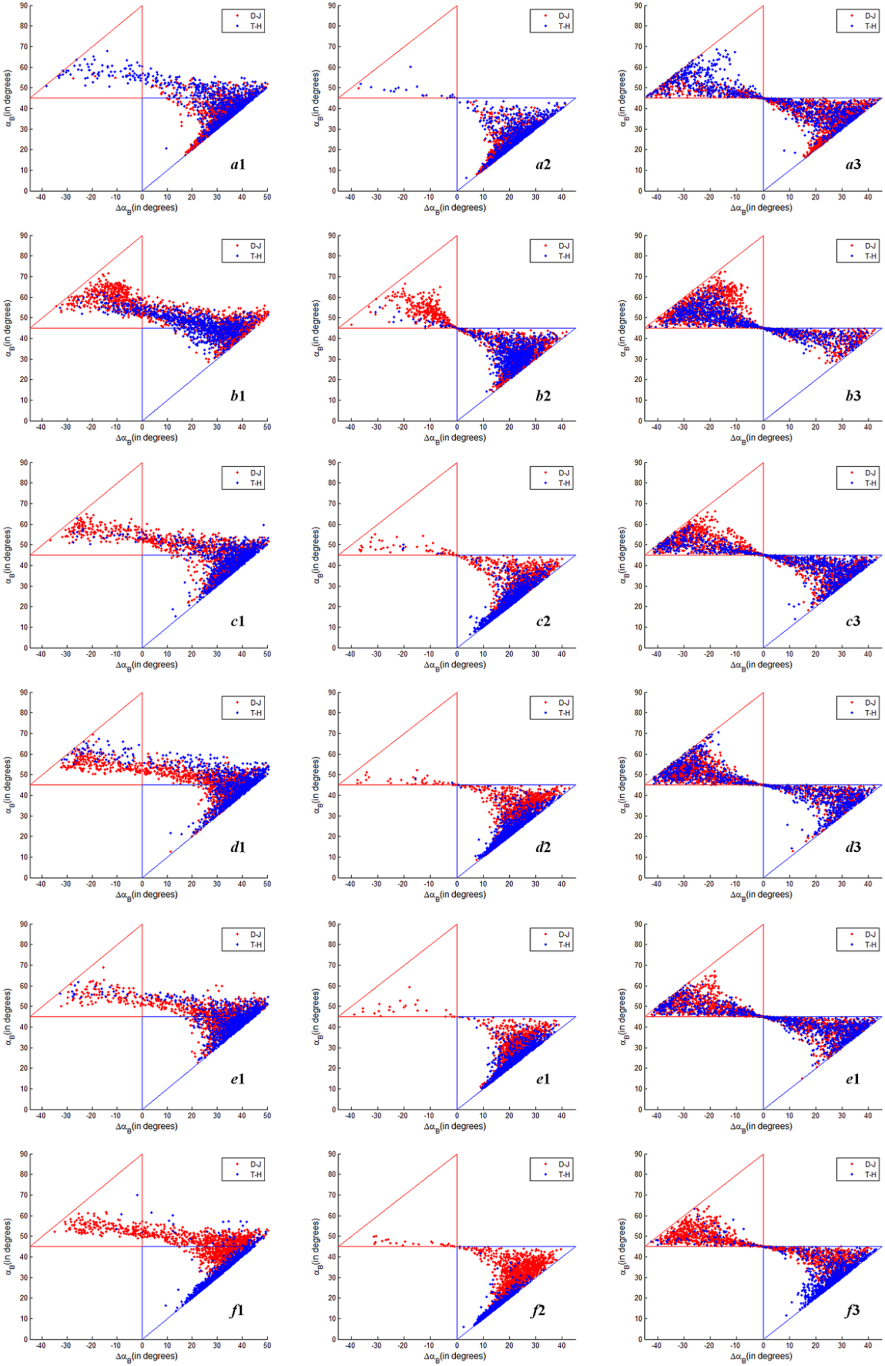
Δα*_B_* and α*_B_* parameters scatter diagram of T-H and D-J based on FP SAR data, CP SAR data of π/4 mode and CTLR mode (The x-coordinate is Δα*_B_* and the y-coordinate is α*_B_*; *a*_1_, *b*_1_, *c*_1_, *d*_1_, *e*_1_ and *f*_1_ are α*_B_* and Δα*_B_* scatter diagram of T-H and D-J based on FP SAR data on June 12, July 30, August 23, September 16, October 10 and November 3 respectively; *a*_2_, *b*_2_, *c*_2_, *d*_2_, *e*_2_ and *f*_2_ are α*_B_* and Δα*_B_* scatter diagram of T-H and D-J based on CP SAR data of π/4 mode on June 12, July 30, August 23, September 16, October 10 and November 3 respectively; *a*_3_, *b*_3_, *c*_3_, *d*_3_, *e*_3_ and *f*_3_ are α*_B_* and Δα*_B_* scatter diagram of T-H and D-J based on CP SAR data of CTLR mode on June 12, July 30, August 23, September 16, October 10 and November 3 respectively.).

In order to analyze the discrimination ability of six temporal Δ*α_B_* and *α_B_* parameters based on FP SAR data, CP SAR data of π/4 mode and CTLR mode to distinguish between the two types of rice paddies in detail, we extract the Δ*α_B_* and *α_B_* parameter values of T-H and D-J training areas, and draw the scatter diagram. **Figure 7** shows *α_B_* and *α_B_* parameters scatter diagram of T-H and D-J based on FP SAR data, CP SAR data of π/4 mode and CP SAR data of CTLR mode on June 12, July 30, August 23, September 16, October 10 and November 3. As can be seen from **Figures 7A–C** on June 12, July 30 and August 23, three kinds of SAR data based on Δ*α_B_* and *α_B_* methods cannot effectively distinguish D-J and T-H. And the Δ*α_B_* and *α_B_* of D-J and T-H are confused on the scatter diagram.

**Figures 7D, E** show Δ*α_B_* and *α_B_* parameters scatter diagrams of T-H and D-J based on FP SAR data, CP SAR data of π/4 mode and CTLR mode on September 16 and October 10. Compared with **Figure 7** *d*_1_, *d*_2_ and *d*_3_, we can find that Δ*α_B_* and *α_B_* of **Figure 7** *d*_2_ are better than **Figure 7** *d*_1_ and *d*_3_ in distinguishing the two types of rice paddy. That is to say, on September 16 (Heading–Flowering stage), CP SAR data of π/4 mode was better than FP SAR data and CP SAR data of CTLR mode in distinguishing the two types of rice paddies. However, on October 10 (Dough–Mature stage), the rice ear of T-H and D-J have basically grown well, and their scattering characteristics are similar. As shown in **Figure 7** *e*_1_, *e*_2_ and *e*_3_, Δ*α_B_* and *α_B_* of two types of rice paddies in this period are not as different as those of September 16. **Figure 7** *f* shows Δ*α_B_* and *α_B_* parameters scatter diagram of T-H and D-J based on FP SAR data, CP SAR data of π/4 mode and CTLR mode on November 3. As shown in **Figure 7** *f*_1_, *f*_2_ and *f*_3_, Δ*α_B_* and *α_B_* of the two types of rice paddies are significantly different. And in the ordinate (*α_B_*), *α_B_* based on CP SAR data of π/4 mode is better than FP SAR data and CP SAR data of CTLR mode. As shown in **Figure 7** *f*_2_, the *α_B_* value of T-H is between 5° and 25°, and the *α_B_* value of D-J is between 25° and 50°. The two types of rice paddies can be well distinguished by this parameter. For *α_B_* based on FP SAR data and CP SAR data of CTLR mode, although *α_B_* value of most D-J is larger than T-H, there is confusion. Regarding Δ*α_B_*, the values based on FP SAR data and CP SAR data of CTLR mode range from -40° to 50°, which is a broader range than that observed for Δ*α_B_* based on CP SAR data of π/4 mode. In terms of the discrimination effect, the ability of *α_B_* based on FP SAR data and CP SAR data of CTLR mode to distinguish between the two types of rice paddies is superior to that of CP SAR data of π/4 mode.

Overall, Δ*α_B_* and *α_B_* showed the best performance in distinguishing between the two types of paddies on November 3, which is closely related to the differences of scattering characteristics between the two types of paddies during this phenological stage. The second-best performance was observed with *α_B_* based on CP SAR data of π/4 mode on September 16, which reflects the differences of rice ear between the two types of paddies. However, Δ*α_B_* and *α_B_* based on FP SAR data and CP SAR data of CTLR mode showed no difference between the two types of rice paddies on September 16. For the Δ*α_B_* and *α_B_* parameters in other periods, the distinction between the two types of rice paddies is not obvious.

### 4.2 Δ*α_B_* and *α_B_* parameters analysis of two types of rice paddies based on general CP SAR

In section 4.1, we conducted a differential analysis of Δ*α_B_* and *α_B_* parameters of two types of rice paddies based on FP SAR data, CP SAR data of π/4 mode and CTLR mode in six phenological periods. To explore CP SAR differences of two types of rice paddies under arbitrary transmit wave, we calculated six temporal Δ*α_B_* and *α_B_* parameters of two types of rice paddies based on general CP SAR data (Section 3.1.2). Based on the theory of general CP descriptors in Section 3.1.2, for a fixed scattering matrix *S*, the widely accepted CP signal depends entirely on *θ* and *χ* (or *a* and *b*). For the two parameters, *χ* ranged from -π/4 to π/4, and *θ* ranged from -π/2 to π/2. For linear π/4, left circular, right circular, the horizontal and vertical polarization transmit wave, the values of $\left(\begin{array}{cc} \theta , & \chi \end{array}\right)$ correspond to $\left(\begin{array}{cc} \text{π}/4, & 0 \end{array}\right)$ , $\left(\left[\begin{array}{cc} -\text{π}/2 & \text{π}/2 \end{array}\right]\begin{array}{cc} , & \text{π}/4 \end{array}\right)$ , $\left(\begin{array}{cc} \left[\begin{array}{cc} -\text{π}/2 & \text{π}/2 \end{array}\right], & -\text{π}/4 \end{array}\right)$ , $\left(\begin{array}{cc} 0, & 0 \end{array}\right)$ and $\left(\begin{array}{cc} \text{π}/2, & 0 \end{array}\right)$ respectively. Circular polarization is not affected by wave orientation angle, so $\theta \in \left[\begin{array}{cc} -\pi /2 & \pi /2 \end{array}\right]$ .

**Figure 8:**
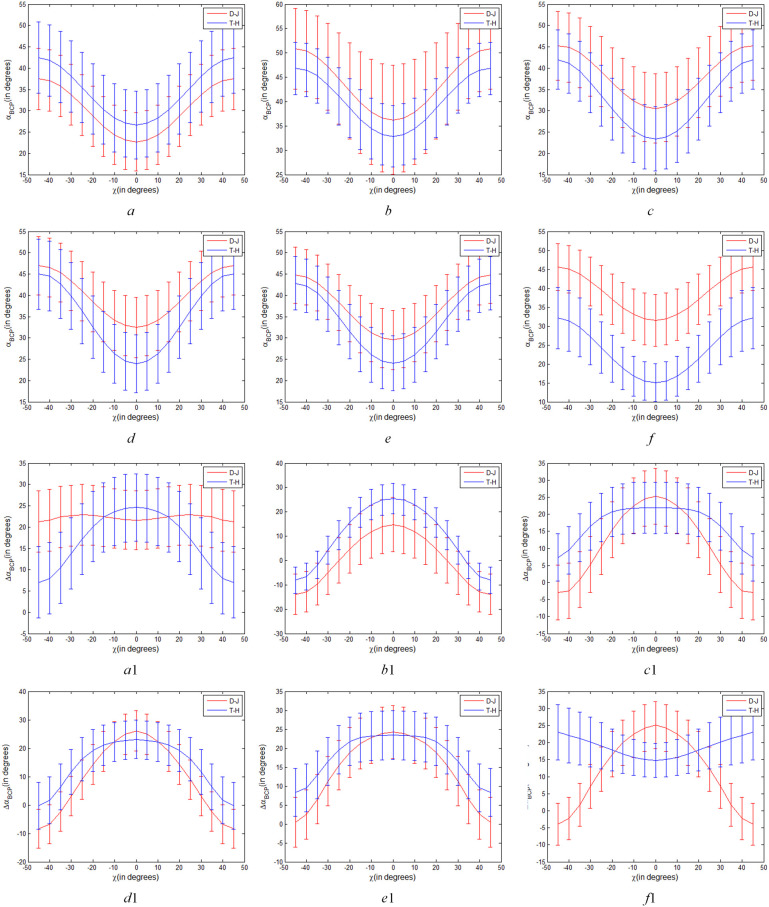
Variations of *α_B_* and Δ*α_B_* parameters of T-H and D-J for varying transmitting polarizations (a fixed *θ*=π/4 and variable $\chi \in \left[\begin{array}{cc} -\pi /4 & \pi /4 \end{array}\right]$ ), **(A–F)** are variations of *α_B_* parameter on June 12, July 30, August 23, September 16, October 10 and November 3 respectively. *a*_1_ *-f*_1_ are variations of Δ*α_B_* parameter on June 12, July 30, August 23, September 16, October 10 and November 3 respectively).

**Figure 9:**
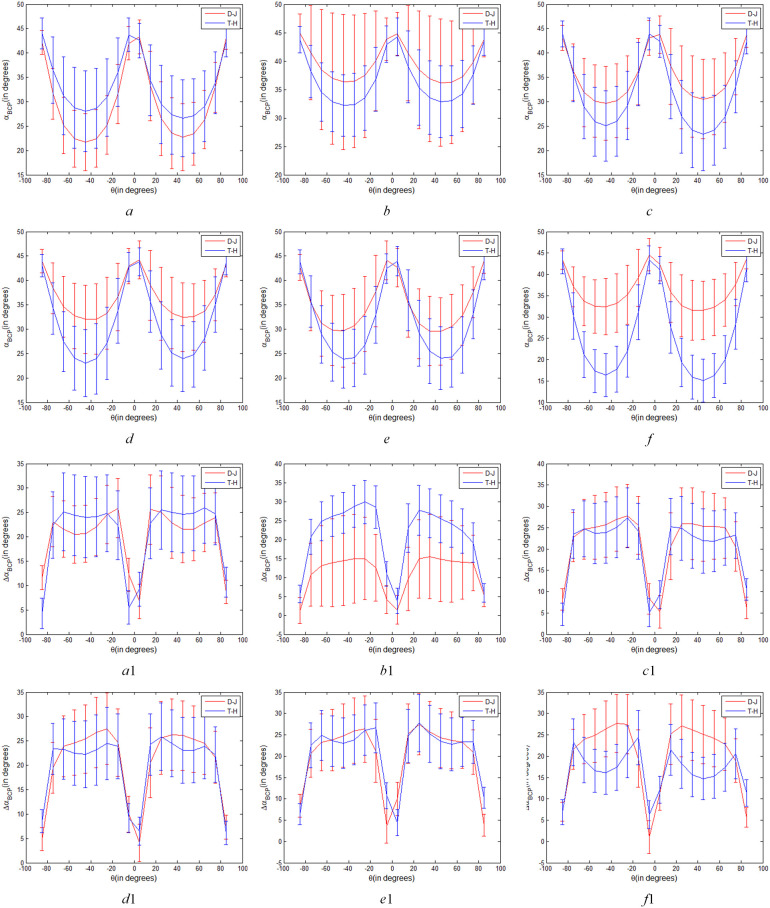
Variations of *α_B_* and Δ*α_B_* parameters of T-H and D-J for varying transmitting polarizations (a fixed χ =0 and variable $\theta \in \left[\begin{array}{cc} -\pi /2 & \pi /2 \end{array}\right]$ ), (*a, b, c, d, e* and *f* are variations of α*_B_* parameter on June 12, July 30, August 23, September 16, October 10 and November 3 respectively. *a*_1_, *b*_1_, *c*_1_, *d*_1_, *e*_1_ and *f*_1_ are variations of Δ*α_B_* parameter on June 12, July 30, August 23, September 16, October 10 and November 3 respectively).

Therefore, we set one variable of *θ* and *χ* (or *a* and *b*) unchanged and change the other variable to extract six temporal Δ*α_B_* and *α_B_* parameters of two types of rice paddies, so as to explore the difference of two types of rice paddies under arbitrary transmitting mode of CP. **Figure 8** shows the curves of *α_B_* and Δ*α_B_* parameters of T-H and D-J for varying transmitting polarizations (a fixed *θ*=π/4 and variable $\chi \in \left[\begin{array}{cc} -\pi /4 & \pi /4 \end{array}\right]$ ) respectively. And, **Figure 9** show the curves of *α_B_* and Δ*α_B_* parameters of T-H and D-J for varying transmitting polarizations (a fixed *χ*=0 and variable $\theta \in \left[\begin{array}{cc} -\pi /2 & \pi /2 \end{array}\right]$ ) respectively.

As shown in **Figures 8A–F**, the values of *α_B_* of T-H and D-J decreased significantly with *χ* approaching 0. However, as shown in **Figures 8A, B** on June 12, July 30, with the change of *χ*, difference value of *α_B_* of T-H and D-J showed little difference. These results indicated that CP SAR of different polarization modes shows little difference in *α_B_* characterization between the two types of rice paddies at seedling stage and initial growth stage. However, from August 23 to November 3, when *χ* is close to 0°, the *α_B_* values of T-H and D-J are significantly different, and when *χ*=0°, the difference was the largest. Since *θ*=π/4 is fixed, the closer to the linear π/4 mode, the more obvious the difference between the two types of rice paddies, especially September 16 and November 3. In accordance with the analysis results in Section 4.1, from August 23 to November 3, the linear π/4 mode of *α_B_* is better than the circular and elliptic polarization modes for discriminating between the two types of rice paddies.

Different from **Figures 8A–F**, it can be seen from **Figure 8** *a*_1_-*f*_1_that the difference value of Δ*α_B_* of T-H and D-J changed with the change of *χ*. For example, in **Figure 8** *f*_1_, when *χ* is ±π/4, corresponding to the circular polarization mode, the difference in Δ*α_B_* between T-H and D-J is most pronounced. As the absolute value of *χ* decreases, the difference value of Δ*α_B_* of T-H and D-J also decreases, indicating that the Δ*α_B_* parameter of circular polarization mode can show the difference of T-H and D-J better than that of elliptic polarization mode. As the absolute value of *χ* continues to decrease, when it is closer to 0, that is to say, closer to the linear polarization mode, the difference between T-H and D-J gradually becomes larger. This indicates that the circular polarization mode and linear π/4 mode show the most obvious differences between the two types of rice paddies in Harvest stage (on November 3).

As shown in **Figures 9A–F**, when *χ* is 0, that is, CP polarization mode was linear polarization mode, *θ* changed from -π/2 to π/2, *α_B_* parameter of T-H and D-J changed significantly. As shown in **Figures 9A–F**, *α_B_* parameter has the same variation trend in different stages. When *θ* is ±π/4 (linear π/4 mode), the difference value between the two types of rice paddies is most obvious compared with other linear polarization modes. Combined with **Figures 8A–F**, it can be seen that the *α_B_* of the linear π/4 mode in six phenological periods is obviously better than that of the other mode in distinguishing between the two types of rice paddies.

### 4.3 Rice paddy classification based on SVM method using Δ*α_B_* and *α_B_* parameters

**Figure 10:**
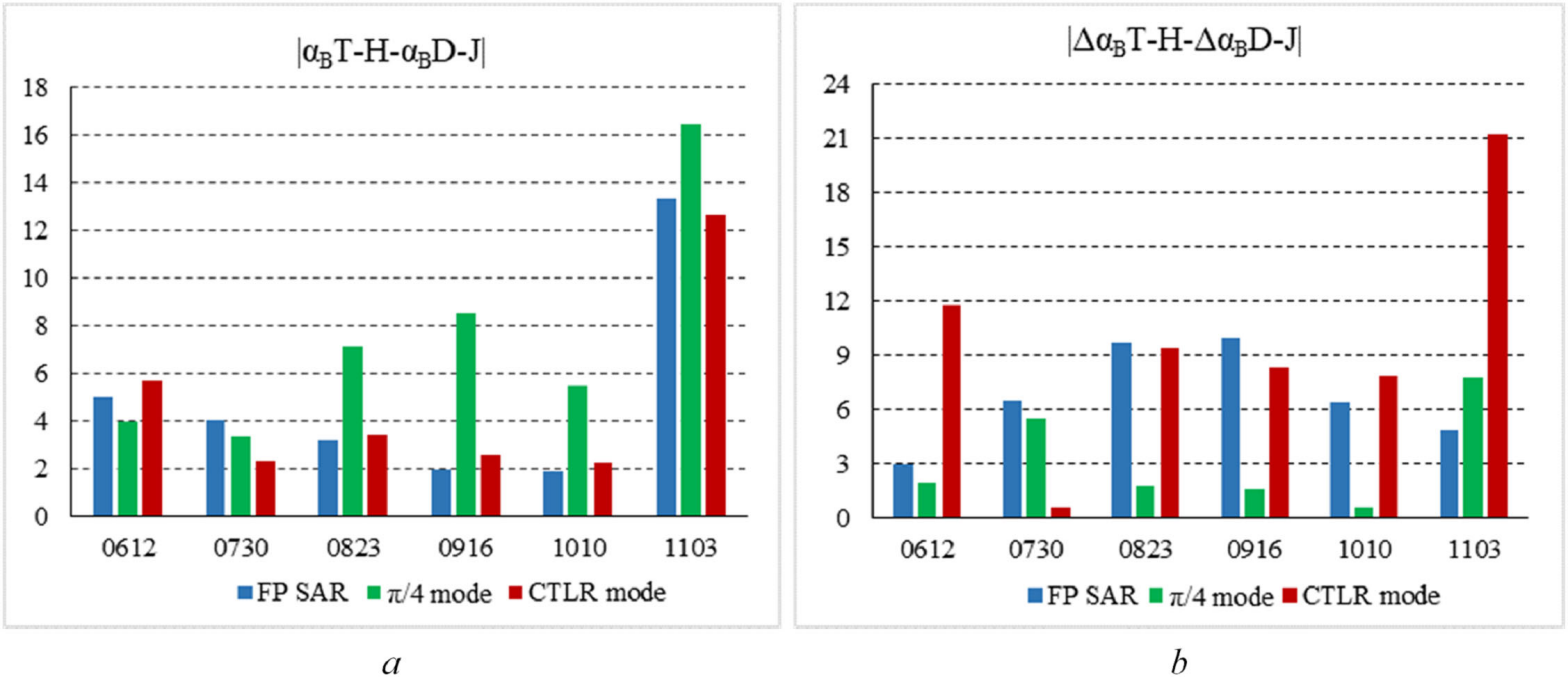
Difference histogram for T-H and D-J of Δ*α_B_* and *α_B_* based on FP SAR data, CP SAR data of π/4 mode and CTLR mode respectively **(A)** is difference histogram for T-H and D-J of α_B_; **(B)** is difference histogram for T-H and D-J of Δ*α_B_*).

To quantitatively evaluate six temporal Δ*α_B_* and *α_B_* parameters in distinguishing between T-H and D-J, as shown in **Figure 10**, we made the difference histogram for T-H and D-J of Δ*α_B_* and *α_B_* based on FP SAR data, CP SAR data of π/4 and CTLR modes respectively.

As shown in **Figure 10A** that difference degree for T-H and D-J of *α_B_* is greater on November 3 than in other periods. Besides, the difference degree for T-H and D-J of *α_B_* based on CP SAR data of π/4 mode is bigger than that based on FP SAR data and CP SAR data of CTLR mode on August 23, September 16, October 10 and November 3. Compared with other periods, the difference degree for T-H and D-J of *α_B_* on November 3 was the largest. Therefore, compared with other periods of *α_B_*, *α_B_* is the best parameter to distinguish between two types of rice paddies, which is consistent with the conclusions of the analysis in section 4.1. As shown in **Figure 10B** that difference degree for T-H and D-J of Δ*α_B_* based on CP SAR data of CTLR mode is greater on November 3 than in other periods. Additionally, in other periods, the difference degree based on FP SAR and CP SAR data of CTLR mode is similar, which is larger than the difference degree based on CP SAR data of π/4 mode.

**Figure 11:**
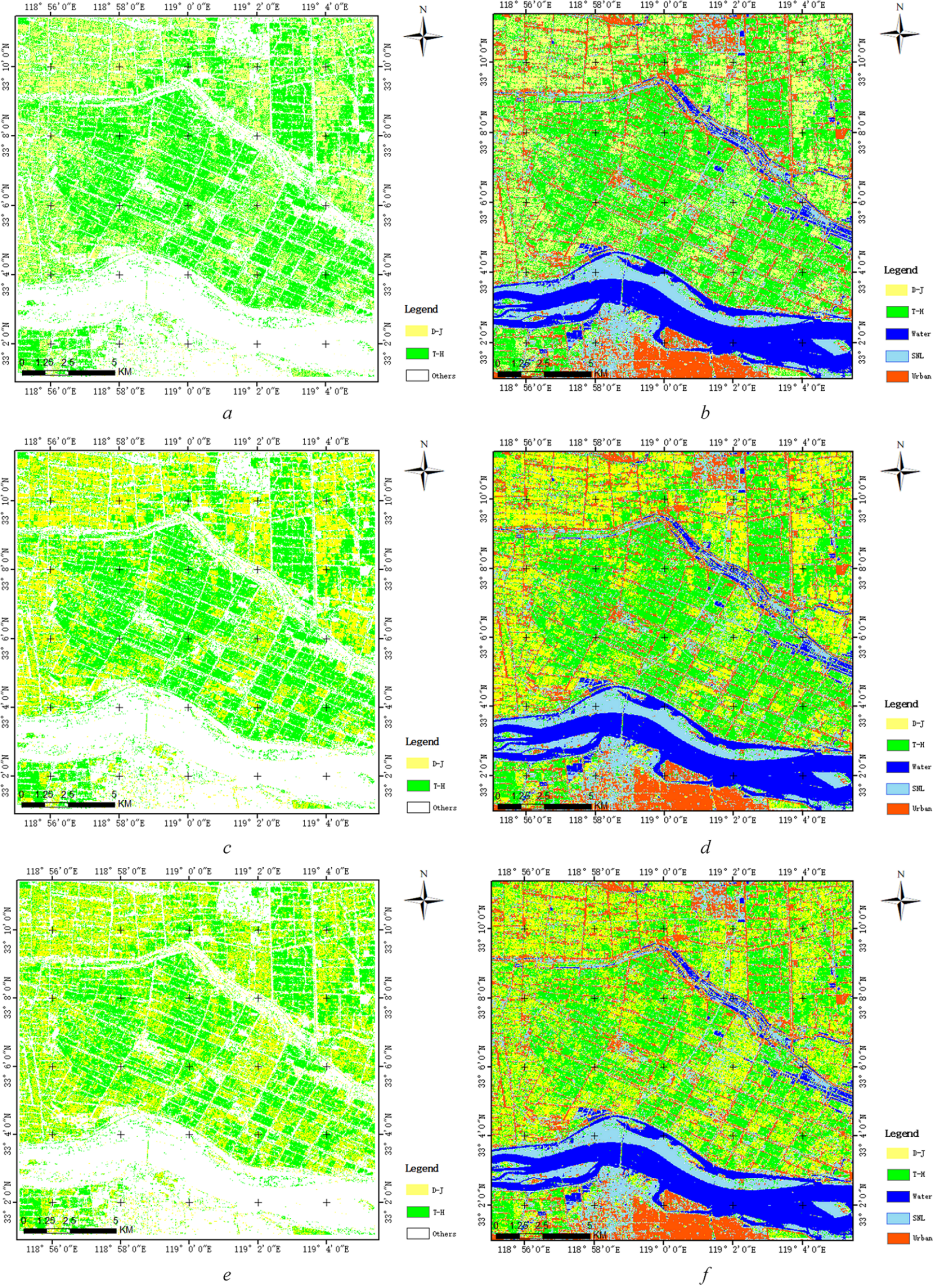
Classification result **(A)** is classification result of T-H and D-J and **(B)** is the overall classification result including 5 classes based on FP SAR data; **(C)** is classification result of T-H and D-J and **(D)** is the overall classification result including 5 classes based on CP SAR data of π/4 mode; **(E)** is classification result of T-H and D-J and **(F)** is the overall classification result including 5 classes based on CP SAR data of CTLR mode).

As shown in **Table 2**, we selected the optimal parameters of Δ*α_B_* and *α_B_* for distinguishing two types of rice paddies based on difference degree of **Figure 10**. We used the optimal parameters of Δ*α_B_* and *α_B_* under three kinds of SAR data to carry out the SVM classification to realize the fine classification of two types of rice paddies. **Figure 11** shows the classification results based on FP SAR data, CP SAR data of CTLR mode and π/4 mode, respectively. As can be seen from the classification results, towns and cities are mostly distributed in the south of the study area, rivers in the middle of the study area, and SNL classes are mostly distributed on both sides of the river. In addition, D-J is mostly distributed in the northwest of the study area. And T-J is mostly distributed in the southeast. The classification results were consistent with the actual distribution of rice cultivation in the study area. Compared with the three classification results, the classification results are generally consistent, but there are differences in details. To better evaluate the three classification results, we used the validation data to verify the classification results.

**Table 2:** The optimal parameters of Δ*α_B_* and *α_B_* for distinguishing two types of rice paddies.

DATA	Optimal parameters
**FP SAR**	α_B__1103, α_B__0612, α_B__0730, Δα_B__0823, Δα_B__0823, Δα_B__0916, Δα_B__1010, Δα_B__0730
**CP SAR data of π/4 mode**	α_B__1103, α_B__0916, α_B__0823, α_B__1010, α_B__0612, α_B__0730, Δα_B__1103, Δα_B__0730
**CP SAR data of CTLR mode**	α_B__1103, α_B__0612, α_B__0823, Δα_B__1103, Δα_B__0612, Δα_B__0823, Δα_B__0916, Δα_B__1010

**Table 3** shows the accuracy indexes of the classification results based on FP SAR data, CP SAR data of CTLR mode and π/4 mode, respectively. This study focuses on distinguish two types of rice paddies based on Δ*α_B_* and *α_B_* parameters, so we discuss only the classification accuracy of the two types of rice paddies and the overall accuracies of the classification results. By comparing accuracy of classification results based on FP SAR data, CP SAR data of CTLR mode and π/4 mode, classification of rice paddy using CP SAR data of π/4 mode shows the best classification results with overall accuracy of 95.5% and kappa of 0.938. Based on Δ*α_B_*/*α_B_* target decomposition method, the classification result using CP SAR data of π/4 mode is higher than that of using FP SAR data and CP SAR data of CTLR mode. Specifically, the overall accuracy of CP SAR data of π/4 mode is 4% higher than that of using CTLR mode, and the Kappa coefficient is greater than 0.05. For rice paddy, in the classification results based on CP SAR data of π/4 mode, the average accuracy of T-H and D-J is 80.9% and 90.0%, respectively. In the classification results based on CP SAR data of CTLR mode, the average accuracy of T-H and D-J is 63.8% and 75.4%, respectively. In the classification results based on FP SAR data, the average accuracy of T-H and D-J is 77.7% and 86.0% respectively. Therefore, we can see that the classification results using CP SAR data of π/4 mode are better than those of using CP SAR data of CTLR mode, and the results are similar to those of using FP SAR data.

**Table 3:** Accuracy table of classification based on FP SAR data.

Method	Class	PA %	UA %	Average accuracy	OA %	Kappa
Classification based on FP SAR data	Water	99.85	100.0	99.92	94.58%	0.925
	Unban	98.75	98.84	98.79		
	SNL	97.70	91.78	94.74		
	T-H	77.65	77.77	77.71		
	D-J	83.01	88.97	85.99		
Classification based on CP SAR data of π/4 mode	Water	99.85	100.0	99.92	95.51%	0.938
	Unban	98.75	98.97	98.79		
	SNL	97.71	91.73	94.74		
	T-H	76.41	85.53	77.71		
	D-J	91.07	88.92	85.99		
Classification based on CP SAR data of CTLR mode	Water	99.85	100.0	99.92	91.62%	0.884
	Unban	98.75	98.12	98.43		
	SNL	97.71	91.71	94.71		
	T-H	61.86	65.70	63.78		
	D-J	74.32	76.41	75.36		

### 4.4 Phenological analysis of Δ*α_B_* and *α_B_* parameters of T-H and D-J

**Figure 12:**
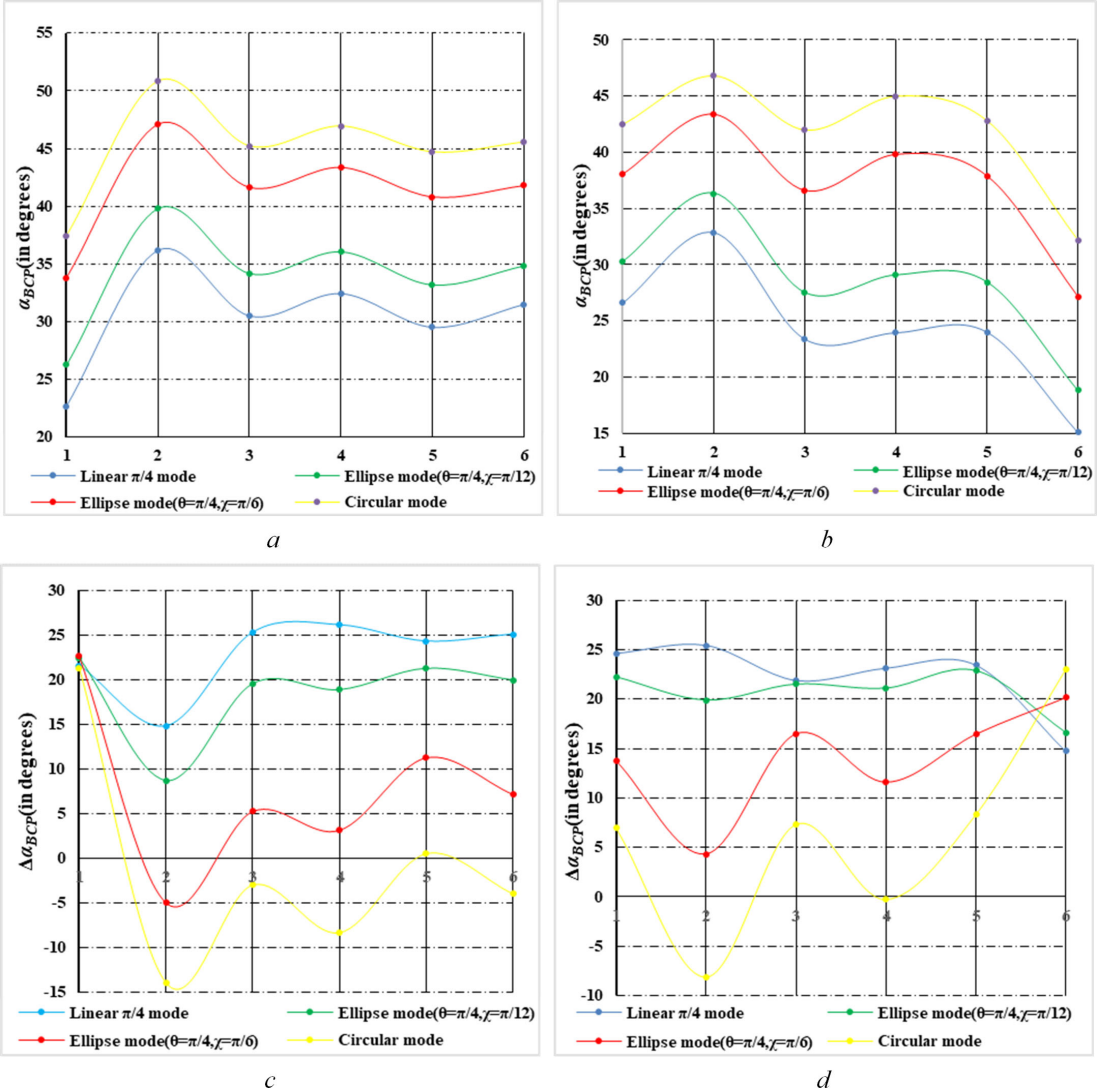
Variations of *α_B_* and Δ*α_B_* parameter of rice paddy for four transmitting polarization modes in six phenological periods **(A, B)** are variations of *α_B_* parameter of D-J and T-H respectively. **(C, D)** and *d* are variations of Δ*α_B_* parameter of D-J and T-H respectively. And the ordinates 1-6 represent 6 phenological periods (on June 12, July 30, August 23, September 16, October 10 and November 3).

With the growth of rice plants, rice morphology will be different under different phenological periods, which results in different expressions of CP parameters at different phenological periods. Therefore, it is of great significance to analyze the CP parameters under different phenological periods for rice phenological recognition. In this section, we analyzed the Δ*α_B_* and *α_B_* of four CP modes of general CP SAR in multiple phenological periods of two types of rice paddies respectively, so as to obtain the change rule of Δ*α_B_* and *α_B_* of two types of rice paddies in the phenological periods under multiple CP modes. **Figures 12A, B** show variations of *α_B_* parameter of rice paddy (T-H and D-J) for four transmitting polarization modes in six phenological periods. In the seedling stage, the vegetation is small and the scattering component is mainly surface scattering. The *α_B_* parameter values of both kinds of rice paddy are low. Due to the different planting methods and varieties of rice, the scattering component of T-H is more abundant than that of D-J in seedling stage, so the *α_B_* of T-H is larger than that of D-J. With increasing growth of rice plants, both plants have increased volume and double-bounce scattering, resulting in greater *α_B_* value of T-H and D-J at elongation stage than at seedling stage. As the rice ear grows, the volume scattering of rice continues to increase. Since the plant is denser, this results in a lower amount of double-bounce scattering. Therefore, at the Booting stage, *α_B_* of T-H and D-J decreased slightly. As rice ears continue to grow from heading stage to mature stage, the scatterings of T-H and D-J tend to be stable, and *α_B_* do not change significantly. Until the Harvest stage, the growth cycle of D-J is longer than that of T-H, so on November 3, T-H has been harvested, while D-J is still in the mature stage and has not been harvested. Therefore, during this period, the *α_B_* of T-H has a significant decrease compared with October 10. However, D-J has no such trend in Harvest stage (November 3). Therefore, the variation trend of *α_B_* parameters of two types of rice paddies is different under the phenological period of rice growth due to the difference of two types of rice varieties.

For different transmitting polarization modes, *α_B_* parameters gradually increase from linear polarization to circular polarization mode, which is similar to **Figures 8A–F** results are consistent. In addition, the variation trend of *α_B_* parameters of T-H and D-J is basically the same for different transmitting modes, which could show the variation rule of the CP parameters in each phenological period.

**Figures 12C, D** show variations of Δ*α_B_* parameter of rice paddy (T-H and D-J) for four transmitting polarization modes across six phenological periods. In the seedling stage, the vegetation is small and the scattering component is mainly surface scattering. The Δ*α_B_* parameter values of the two types of rice paddies are greater than 0. As rice grows into Elongation stage, the double-bounce scattering increases. Therefore, the Δ*α_B_* of T-H and D-J at Elongation stage is lower than that at seedling stage. From October 10 to November 3, the changes of Δ*α_B_* of T-H and D-J were different because T-H was harvested on November 3, while D-J was still in the mature stage. As a result, the variation trends of Δ*α_B_* are quite different. In addition, the variation trend of Δ*α_B_* curves for T-H and D-J shows that Δ*α_B_* under the circular mode exhibits a much more obvious difference in the characteristics of each phenological period of rice compared to other modes.

In general, there was a certain difference in the variation trend of *α_B_* parameters between the two types of rice paddies with the growth of rice plants. From Seedling to Elongation stage, the variation trend of the T-H and D-J is basically the same, but the degree of change differs, which is related to the planting methods and varieties of the two types of rice. However, from mature stage to harvest stage, the variation trend of *α_B_* parameters differs significantly, mainly due to the length of growth cycle of the two types of rice. For the Δ*α_B_* parameter, with the growth of rice plant, Δ*α_B_* changes dramatically, and the variation trends of the two types of rice differ. Taking the circular mode as an example, the most obvious difference is from mature stage to harvest stage, and the Δ*α_B_* parameter variation trend of the T-H and D-J is just the opposite. In addition, from seedling stage to harvest stage, changes degree of Δ*α_B_* under circular mode were more obvious than other mode, which also showed the difference between the two types of rice paddies.

This paper mainly studied the classification and phenological analysis of rice based on the optimal polarimetric parameters that characterize the scattering characteristics of rice plants. However, environmental factors also affect rice classification and phenological analysis to a certain extent. The specific influencing factors mainly include the following three aspects. 1. Topography factors: The study area is a plain region with flat terrain, so the topography has little impact on this study. However, in mountainous and hilly areas where rice is grown, radar side imaging may cause overlay and shadow effects in paddy fields. Consequently, polarimetric SAR parameters may not effectively characterize rice scattering characteristics in these regions. In future work, we will acquire SAR data of paddy fields in mountainous and hilly areas to study the influence of topography on rice classification and phenological analysis. 2. Soil factors. In SAR images, soil moisture is the main factor affecting polarimetric scattering characteristics of crop. Since our research focuses on rice, the underlying surface environment varies with different rice phenological stages. For example, during the Seedling, Elongation, Booting, Heading, and Flowering stages, the rice fields are completely covered by water. However, in the Milky and Mature stages, the underlying surface is wet soil layer. Therefore, the underlying surface of rice varies across different phenological stages, leading to distinct responses in polarimetric parameters in radar images. For rice phenological analysis, the variations in the underlying surface at different phenological stages of rice lead to improved characterization of these stages by polarimetric parameters. 3. Agricultural Management Factors: Agricultural management also has a certain impact on the classification and phenological analysis of rice. For example, this study distinguishes between two types of rice (T-H and D-J), which differ not only in variety but also in cultivation management practices. T-H rice is sown for transplanting, with row and pier spacing of approximately 30 cm and 15 cm, respectively. In contrast, D-J rice, which is planted directly in paddies, shows a random uniform distribution during the seedling stage. This results in significantly different polarimetric characteristics between the two types of rice, allowing them to be effectively distinguished.

## 5 Conclusion

In this study, we proposed a strategy for fine classification and phenological analysis based on general CP SAR data. Based on FP SAR data and general CP SAR data, Δ*α_B_*/*α_B_* methods were introduced into rice fine classification and phenological analysis of rice paddy under multiple phenological periods. And the main research conclusion has two aspects.

On the one hand, based on Δ*α_B_* and *α_B_* parameters, the fine classification results of rice paddy using FP SAR and CP SAR of data of π/4 mode and CTLR mode were obtained and the three results were verified and evaluated. Additionally, we explored the ability of Δ*α_B_* and *α_B_* parameters to distinguish between the two types of rice paddies across multiple phenological periods and extracted the optimal parameters. We found that the Δ*α_B_* and *α_B_* based on the general CP SAR data on November (Harvest stage) are the best parameters for distinguishing between the two types of rice paddies. Moreover, CP SAR data of π/4 mode is better than CP SAR data of CTLR mode and FP SAR data on September 16 (Heading–Flowering stage) in distinguishing between the two types of rice paddies. Additionally, *α_B_* based on CP SAR data of π/4 mode reflects the difference of rice ear between the two types of paddies in this period. Furthermore, we found that CP SAR of different modes had little difference in *α_B_* characterization between the two types of rice paddies at seedling stage and initial growth stage. However, from August 23 (Booting–Heading stage) to November 3 (Harvest stage), π/4 mode of *α_B_* is better than circular and elliptic polarization mode in discrimination ability for two types of rice paddies. In addition, using SVM classification method based on optimal parameters of Δ*α_B_* and *α_B_*, we get high precision classification results of rice paddy. The experimental results show that the classification accuracy is above 90%, and the Kappa coefficient is above 0.88. The highest accuracy of T-H is 80.9%, and the highest accuracy of D-J is 89.9%. Moreover, classification results using CP SAR data of π/4 mode data are better than those using CP SAR data of CTLR mode data, and the results are similar to those of using FP SAR data.

On the other hand, we studied the phenological evolution rule of the two rice types under general CP modes. There was a certain difference in the variation trend of *α_B_* parameters between the two types of rice paddies with the growth of rice plants. From Seedling to Elongation stage, the variation trend of the T-H and D-J is basically the same, but the degree of change is different. From mature stage to harvest stage, the variation trend of *α_B_* parameters is significantly different for two types of rice paddies, which is mainly due to the length of growth cycle of the two types of rice.

## Data Availability

The original contributions presented in the study are included in the article/supplementary material. Further inquiries can be directed to the corresponding author.
